# Rationale and design of BISTRO: a randomized controlled trial to determine whether bioimpedance spectroscopy-guided fluid management maintains residual kidney function in incident haemodialysis patients

**DOI:** 10.1186/s12882-017-0554-1

**Published:** 2017-04-26

**Authors:** Simon J. Davies, Fergus J. Caskey, David Coyle, Elizabeth Lindley, Jamie Macdonald, Sandip Mitra, Martin Wilkie, Andrew Davenport, Ken Farrington, Indranil Dasgupta, Paula Ormandy, Lazaros Andronis, Ivonne Solis-Trapala, Julius Sim

**Affiliations:** 1 Institute for Applied Clinical Sciences, Keele University, Keele, Staffordshire UK; 2 UK Renal Registry and School of Social and Community Medicine, University of Bristol, Bristol, UK; 3 NIHR Devices for Dignity, Sheffield Teaching Hospitals NHS Foundation Trust, Sheffield, UK; 4 Renal Medicine, Leeds Teaching Hospitals NHS Trust, Leeds, UK; 5 School of Sport, Health and Exercise Sciences, Bangor University, Bangor, North Wales UK; 6 Renal Medicine, Central Manchester University Hospitals NHS Foundation Trust, Manchester, UK; 7 Renal Medicine, Sheffield Teaching Hospitals NHS Foundation Trust, Sheffield, UK; 8 Renal Medicine, Royal Free Hampstead NHS Trust, London, UK; 9 Renal Medicine, East & North Hertfordshire NHS Trust, Hertfordshire, UK; 10 Renal Medicine, Heart of England NHS Foundation Trust, Birmingham, UK; 11 School of Nursing, Midwifery, Social Work and Social Science, University of Salford, Manchester, UK; 12 Health Economics Unit, University of Birmingham, Birmingham, UK; 13 University Hospital of North Midlands, Newcastle Rd, Stoke-on-Trent, Staffordshire ST46QG UK

**Keywords:** Fluid status, Body compostion, Residual kidney function, Haemodialysis, Bioimpedance, Fluid management, Health economics

## Abstract

**Background:**

Preserved residual kidney function (RKF) and normal fluid status are associated with better patient outcomes in incident haemodialysis patients. The objective of this trial is to determine whether using bioimpedance technology in prescribing the optimal post-dialysis weight can reduce the rate of decline of RKF and potentially improve patient outcomes.

**Methods/Design:**

516 pateints commencing haemodialysis, aged >18 with RKF of > 3 ml/min/1.73 m^2^ or a urine volume >500 ml per day or per the shorter inter-dialytic period will be consented and enrolled into a pragmatic, open-label, randomized controlled trial. The intervention is incorporation of bioimpedance spectroscopy (BI) determination of normally hydrated weight to set a post-dialysis target weight that limits volume depletion, compared to current standard practice. Clinicians and participants will be blinded to BI measures in the control group and a standardized record capturing management of fluid status will be used in all participants. Primary outcome is preservation of residual kidney function assessed as time to anuria (≤100 ml/day or ≤200 ml urine volume in the short inter-dialytic period). A sample size of 516 was based upon a cumulative incidence of 30% anuria in the control group and 20% in the treatment group and 11% competing risks (death, transplantation) over 10 months, with up to 2 years follow-up.

Secondary outcomes include rate of decline in small solute clearance, significant adverse events, hospitalization, loss of vascular access, cardiovascular events and interventions, dialysis efficacy and safety, dialysis-related symptoms and quality of life. Economic evaluation will be carried out to determine the cost-effectiveness of the intervention. Analyses will be adjusted for patient characteristics and dialysis unit practice patterns relevant to fluid management.

**Discussion:**

This trial will establish the added value of undertaking BI measures to support clinical management of fluid status and establish the relationship between fluid status and preservation of residual kidney function in incident haemodialysis patients.

**Trial registration:**

ISCCTN Number: 11342007, completed 26/04/2016; *NIHR Portfolio number:* CPMS31766; *Sponsor*: Keele University

## Background

Preservation of residual kidney function (RKF) and achieving normal volume status are recognized as two linked and critically important predictors of survival in haemodialysis patients. The CANUSA study found that each 250 ml of urine per day increased 2-year survival by 36% in peritoneal dialysis patients [1] and in the NECOSAD study complete anuria in haemodialysis (HD) patients increased the relative risk of death 17-fold compared to those with well-preserved kidney function [2]. A more recent US study found a graded association, with the fastest rates of RKF loss being associated with doubling of all-cause mortality [3]. Other reported benefits of RKF include improved wellbeing, better quality of life [4] and less need to remove high fluid volumes during dialysis sessions with reduced risks of intra-dialytic hypotension [5], cardiac stunning [6] and associated mortality risk [7, 8]. This link between RKF, volume management and outcomes is further supported by observational studies indicating that increased risks associated with failing to achieve target weight are bidirectional, implying that both over- and under-hydration are a threat to patients [9].

Given the value of RKF, it is surprising how few clinical trials have focussed on interventions to maintain it as a key benefit to HD patients – the exception being ultrapure water [10], which is now standard care. Worse than this, a frequently applied fluid management strategy is to reduce the post-dialysis target weight until minimal or no anti-hypertensive drugs are required as evidence of adequate control of volume status. Our recent survey of fluid management practice patterns in UK units indicates that this is still being pursued in the majority of units [11], despite the risk it poses to residual kidney function by setting in place a continuing vicious cycle of volume depletion, excessive thirst and high inter-dialytic fluid gains.

The introduction of bioimpedance (BI) technology, such as BI spectroscopy, provides clinicians with the opportunity to break this cycle while avoiding the risk of excessive overhydration. BI gives additional information about body composition available at the bedside [12]. The principle is simple and involves the passing of a low-strength alternating current through the subject’s body to measure the resistance and reactance to flow. These two measures are dependent on the amount of fluid and cell membranes between the electrodes (usually placed on the hand and foot on one side of the body). The measurements are then modelled using information such as the subject’s weight and height to estimate the total volume of fluid in the body and the proportion of this that is within cells or in the extracellular space. In dialysis patients, compared to healthy subjects, the total amount of fluid (intra plus extracellular) can be high or low, but often the latter because of muscle wasting, inflammation or even over-aggressive fluid removal on dialysis. However, if the extracellular fluid is disproportionately high, this is a strong signal for an increased mortality risk [13–15]. For this reason, until now trials conducted to establish the clinical value of BI in setting target weights have focussed on clinical endpoints associated with hypervolaemia, such as high blood pressure, left ventricular mass and pulse- wave pressure or worsening extracellular to intracellular fluid distribution. The results of these interventions have been mixed, and more trials are clearly needed, but the price for achieving lower blood pressure through post-dialytic hypovolaemia was accelerated loss of RKF in one such study [16]. There is also evidence that presence of RKF leads to more stable fluid status without intervention in peritoneal dialysis patients [17]. Thus, BISTRO will establish the potential for BI to add value to fluid management and address the concern that this technology may be being adopted indiscriminately without clear evidence of benefit and a potential risk of harm.

## Methods/Design

### Aim

To determine if incorporation of bioimpedance spectroscopy-derived information on body composition into the setting of the post-dialytic target weight reduces loss of residual kidney function in incident centre-based HD patients, with the potential to improve clinical outcomes, dialysis-related symptoms, hospitalization and survival.

### Trial design

A pragmatic, multicentre, open-label prospective randomized controlled trial comparing current best practice in setting the post-dialytic target weight (control group) with the same assessment guided by serial BI measurements (intervention group). BI readings will be taken in both study groups but the results concealed from the clinical teams and trial participants in the control limb. To minimize performance and information bias, the BI measurements will be taken independently from the fluid assessments by trained nurses but within the previous week (i.e. the last 3 dialysis sessions), usually before sessions.

### Study setting

The study will take place in adult, out-patient haemodialysis centres, both main and satellite units, and their associated inpatient renal units during hospital admissions. Patients admitted for inter-current problems while participating in the trial will remain in the study and be assessed according to randomization. Participating sites will be available on the dedicated trial website, www.keele.ac.uk/bistro.

### Eligibility criteria

**Inclusion criteria**

- Adults aged >18 years within 3 months of commencing centre-based maintenance haemodialysis due to advanced kidney disease CKD stage 5, planned or unplanned, via arteriovenous fistula, graft or central venous catheter (i.e. with or without permanent vascular access)
- Commencing dialysis on any regimen, including having incremental dialysis initiation
- Residual kidney function: Patients who have not yet started, but are about to start, dialysis treatment should have a daily urine volume > 500 ml/day *or* a measured mean urea and creatinine clearance ≥3 ml/min/1.72 m^2^ determined from a 24-h collection. Patients already on dialysis should have a urine volume >500 ml during the short inter-dialytic period *or* a measured mean urea and creatinine clearance ≥3 ml/min/1.72 m^2^, determined from the same timed inter-dialytic urine collections and an average of the post and pre dialysis plasma urea and creatinine concentrations.

**Exclusion criteria**

- Unable or unwilling to give informed consent
- Unable to comply with trial procedures, e.g. collection of urine output
- Likely survival prognosis or planned modality transfer < 6 months
- Subjects with limb amputations when the foot is not accessible *and* it is not possible to take hand to hand measurements

### Intervention

The study intervention is the incorporation of bioimpedance technology-derived information about body composition into the clinical assessment of fluid status of dialysis patients. The study intervention is the use of this additional information, specifically the *normally hydrated weight*, in conjunction with usual clinical judgement to set a target dry weight that is as close to normal at the end of a dialysis session, thus avoiding the risks of over- or under-hydration.

BISTRO is investigator-led with no input into the study design from industry. Based upon clearly defined criteria articulated in the original grant application, 6 device manufacturers were invited to submit proposals to a panel comprising experts, patient and lay representation and independent clinician and National Health Service experts. The Fresenius Body Composition Monitor (Fresenius BCM) was the device selected based on best validated device in the renal population both against gold standard methods (*i.e.* DEXA scanning, deuterium and sodium bromide solution) [18] and in referencing body composition of the dialysis patients to population norms [15, 19]. The BCM was originally CE-marked to Fresenius Medical Care as a Class IIa medical device in 2003; the CE mark was last updated in June 2011 [20].

All participating centres will receive bespoke training at the site visits prior to enrolling patients, which will include interpretation of BI data for use by fluid management assessors and a standardized approach to taking BI measurements by the research nurses. The study team will provide support for data interpretation throughout the study (see responsibilities).

### Outcomes

**Primary Outcome: Preservation of Residual Kidney Function**

- Time to anuria, defined as urine volume ≤100 ml/day or ≤200 ml in the short inter-dialytic period confirmed by a further collection after 2 weeks to exclude temporary illness.

**Secondary Outcomes**

- The rate of decline in kidney function, defined as the slope in decline of the average residual urea and creatinine clearance.
- Significant events, including vascular access failure and associated interventions, cardiovascular events, hospital admissions and death, including long-term legacy effects beyond trial completion using data linkage.
- Objective measures of dialysis efficacy and safety: e.g. inter-dialytic fluid gains, intra-dialytic hypotension, urea-reduction ratios (routine data)
- Patient-reported outcomes, including quality of life: EQ-5D-5 L; [21] SF12 [22], dialysis-related symptoms (Integrated Palliative Care Outcome Scale- Renal, IPOS) www.pos-pal.org, Patient Activation Measure [23], Duke Activity Status index [24], Montreal Cognitive Assessment (MoCA) [25], Client Service Receipt Inventory (CSRI) for Chronic Disease.
- Cost-effectiveness of the intervention, expressed as incremental cost per additional quality-adjusted life year (QALY) gained.

### Participant timeline

**Fig. 1:**
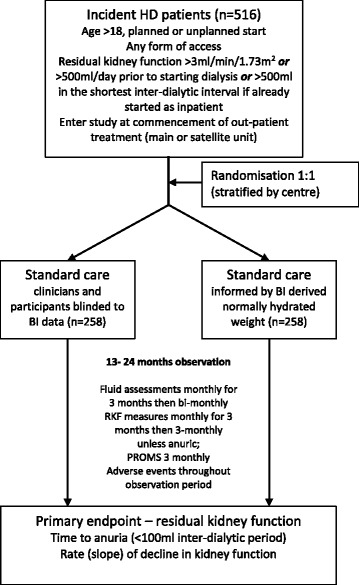
Schema for BISTRO Trial

Trial entry for all participants is at the point of commencing centre-based haemodialysis as an outpatient, see Fig. 1. At this point trial eligibility will be confirmed, followed by randomization. All participants will be followed up until study completion or withdrawal because of death, transplantation, stopping dialysis (e.g. recovery of function) or patient choice, including any period after they reach the primary outcome so that the health economic analysis can be completed. The schedule of trial assessments is shown in Table 1.

**Table 1:** Schedule of visits and procedures

	VISITS (Months) All undertaken at routine dialysis sessions						Urine Collections	Trial completion	Event based
Procedure	Visit −1	BaselineVisit 0	Visit 1	Visit 2	Visit 3	Visits 4–11 At 6,9,12,15,18,21,24 Months.	At 5,7,9,11,13,15,17,19,21,23,24 months. includes extra 2 weeks after primary endpoint is reached		
Eligibility	x								
Consent	x								
Residual kidney function tests for normalized GFR (urine volume and urine + blood to lab)	x		x	x	x		x		
Height (cm)	x								
Web-based randomisation		x							
Date of birth		x							
Ethnicity		x							
Sex		x							
Full medication list		x							
Primary Renal Disease Diagnosis		x							
Stoke comorbidity score		x							
Renal Registry comorbidity fields		x							
Planned/unplanned start		x							
Access type (fistula/graft/line)		x							
HD modality: (HD, HDF)		x							
Incremental/full start dialysis		x							
Transplant wait listed		x							
Dialysis prescription		x							
Bioimpedence with full dataset using software		x	x	x	x	x			x if indicated
BISTRO Study intervention record		x	x	x	x	x			x if indicated
Duke Activity Status Index (ASI)		x			x	x			
Patient Activation Measure (PAM)		x			x	x			
EQ-5D-5 L		x			x	x			
IPOS-Renal patient version		x			x	x			
Haemodialysis symptoms questionnaire		x			x	x			
Short Form (SF-12) Health Survey		x			x	x			
CSRI CKD		x			x	x			
Cognitive Assessment (MoCA)		x					x annually		
Study Termination/completion form								x	x
Adverse events									x

### Sample size

#### Primary outcome (time to anuria)

It can be determined from cohort studies and data collection from a large dialysis unit of 615 patients that the proportion of incident centre-based HD patients anuric by 10 months is in the region of 30% (range 25–67%) [26–30]. Sample size is based on a cumulative 10-month incidence of anuria of 30% in the control group and 20% in the treatment group and 11% competing risks (based on death and transplantation data extrapolated from the 2013 UKRR report [31]). Assuming exponential decline, proportional hazards, 90% power and 5% two-tailed significance, 185 events are required to detect the corresponding hazard ratio, with 12 months accrual and 12 months follow-up. This will require a total of 516 patients to be randomized 1:1, allowing for a 5% loss to follow-up.

#### Secondary outcome

The rate of decline in renal clearance is reported by most studies as a monthly decline of 0.3 ml/min/1.73 m^2^/month (reported range 0.3–0.4) [26–30]. At the same significance level, this sample size would provide just under 95% power to detect a difference in rate of 0.05 ml/min/1.73 m^2^/month, assuming linear change assessments at 0,1,2,3,5,7,9,11 and 13 months, and a (conservative) autocorrelation of 0.30.

### Recruitment

Participants will be recruited over a 12-month period at 30 centre-based haemodialysis centres throughout the UK, including satellite units affiliated with main centres. All adult patients new to centre-based HD treatment will be screened using the trial eligibility criteria. Patients who start dialysis in a planned fashion will be approached in chronic kidney disease clinics at the point of deciding a convenient start date. Patients starting treatment that is unplanned will be approached at the point it is decided they will require long-term dialysis.

### Allocation

Both planned and unplanned incident HD patients will be randomized after informed consent has been obtained and at the point of commencing haemodialysis as an outpatient. Randomization will be 1:1 to the BI intervention and control groups, with random permuted blocks, stratified by centre (main or satellite where dialysis will commence.

Randomization will be during office hours using a secure centralized web-based, automated computer-generated randomization system provided by the Keele University Clinical Trials Unit (CTU). Authorized personnel at the trial site will be allocated personalized log-in details by Keele CTU to access this system. In the event the centre network is not functioning they will call the Keele CTU and authorized staff will perform the randomization on the centre’s behalf.

### Blinding

BI readings will be taken in both study groups but the results concealed from the clinical teams and patients in the controls. To ensure blinding to BI data in control subjects and to minimize performance bias, BI measurements will be taken independently from clinical fluid assessments by the research nurse. The full BI dataset will be stored for all patients using the proprietary software, but in the BI intervention arm the key BI metrics used for informing the clinical decision will be transferred to the clinical assessment CRF (BISTRO study intervention record) prior to their use.

### Data collection: methods and management

#### Demographic data, clinical and bioimpedance assessments of fluid status

**Fig. 2:**
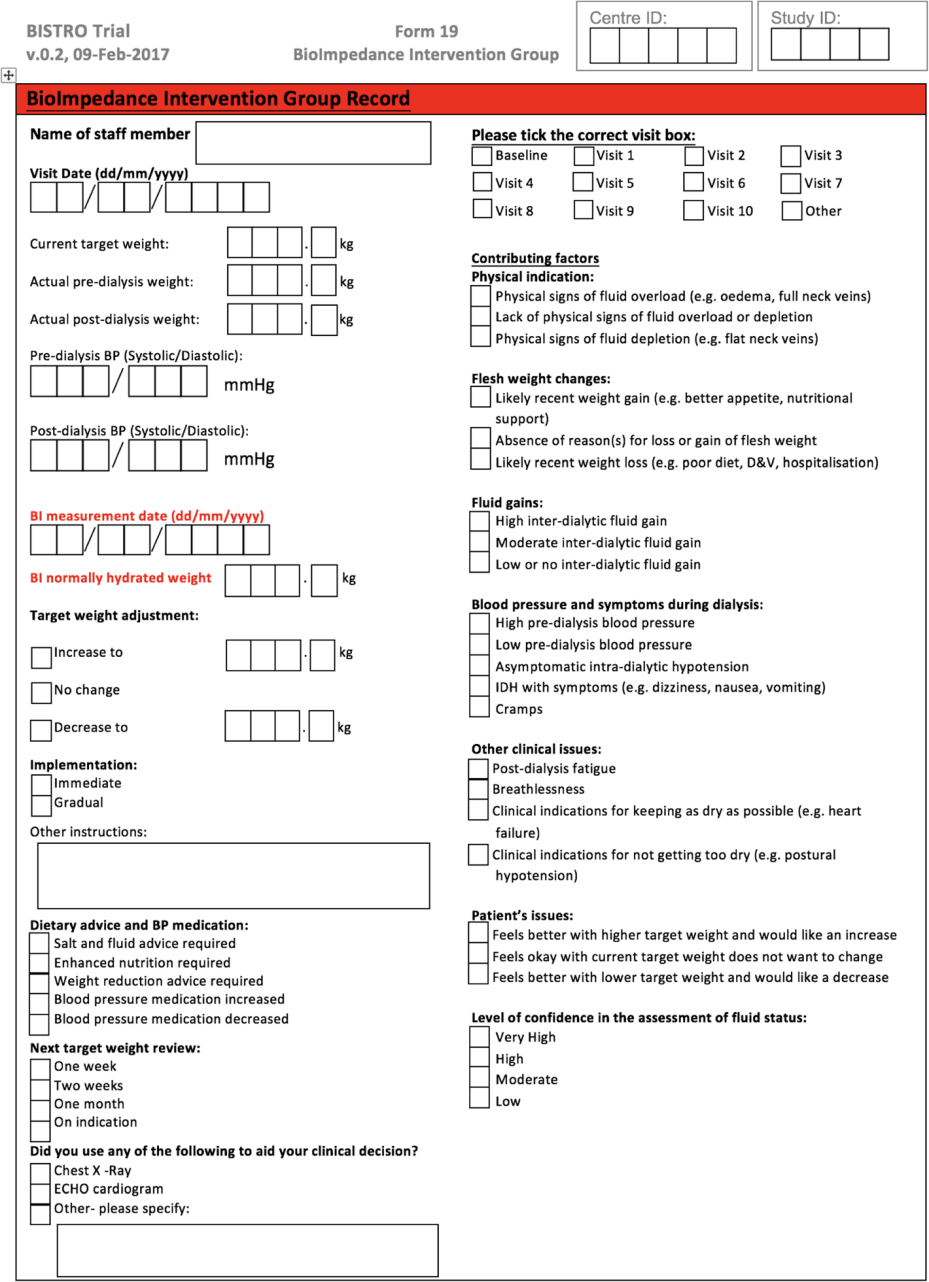
BISTRO fluid assessment record. At each fluid assessment the clinician will complete this chart, recording any target weight adjustment, planned interventions to achieve this and any factors that have influenced their decision to set or override the suggested target weight. Only in the those patients randomised to the intervention will the normally hydrated weight post dialysis be completed and thus available to the clinician

Data at baseline and subsequently (see Table 1) will be collected on paper CRFs at the specified time intervals – with the option of recording additional fluid assessment if clinically indicated (e.g. following hospital admission). Fluid assessments are recorded on the BISTRO study intervention record, onto which the BI-determined normally hydrated weight is transferred in the intervention group only (see Fig. 2). Electronic capture of a full bioimpedance dataset is supported by the device software and stored on the dialysis unit server for later transfer to the CTU.

#### Residual kidney function

This is determined from a urine collection and routinely collected blood samples for urea and creatinine. The research nurse will enter these data into a GFR calculator to calculate residual kidney function. The whole procedure will be carried out in accordance with a study-specific standard operating procedure for measurement of residual renal function in haemodialysis patients by the Leeds Teaching Hospitals NHS Trust and adapted for the BISTRO trial.

#### Patient reported outcomes

These will be administered using a patient booklet containing the items listed in Table 1. The Montreal Cognitive Assessment (MoCA) will be administered by research nurses who have received training.

#### Unit-level survey of practice patterns

A unit-level survey will be completed annually for the project duration by the Lead Consultant for each dialysis Unit (see Table 2 for items).

**Table 2:** Unit level practice patterns, measured annually: first completion just before the first patient is enrolled

Dialysate sodium concentration
• Is there a standard sodium concentration in your unit? • What is the concentration of sodium used most frequently? • What proportion of patients have an individualised sodium concentration? • If individual sodium used o If low, what reason? If high, what reason? o Is your practice to match the plasma sodium?
Nutrition and sodium intake
• Does your HD unit have a dedicated dietitian? If so, how much time per patient do they have? • Do you have a policy on sodium restriction? If so what is the advised intake? • Do you have a policy on fluid restriction? If so what is the advised intake? • What information/training is given to nurses on the HD unit about fluid/salt restrictions? • Are patients given written advice about dietary intake and restrictions?
Diuretics
• Are the majority patients with residual kidney function routinely prescribed loop diuretics? • What is the typical dose (e.g. Furosemide, Bumetanide)? • Do you routinely use other diuretics (metolazone, thiazides, aldosterone inhibitors)?
Incremental dialysis
• Is it routine practice in your unit to commence HD incrementally? • If so, is this to preserve residual kidney function? • What proportion of patients on your unit do (a) 1 or (b) 2 sessions per week in the context of incremental start?
Measurement of residual Kidney Function
• Do you routinely measure residual kidney function on your unit? If so how frequently? • If so, do you use this to reduce the (a) frequency, (b) length of dialysis sessions?
Assessment and prescription
• Do you have a standardised protocol for assessing fluid status in new HD patients? • Protocol or not, in addition to clinical assessment do you routinely use (a) bioimpedance – of so state device, (b) Chest Xray,(c) Echocardiogram (d) central vein diameter, (e) blood volume monitoring? • Who assesses fluid status on your unit (a) consultants (b) HD dedicated staff grades (c) HD nurses (d) training grade doctors.
Fluid management strategies
• Who prescribes fluid management on your unit (a) consultants (b) HD dedicated staff grades (c) HD nurses (d) training grade doctors. • Do you have a policy to maximise UF rates in your unit? If so, what is the maximum rate permitted? • If you are changing the target weight, typically what is the maximum change per session you would prescribe? (Exclude urgent situations and tell us if there is no specific policy on this).

#### Routine clinical data collected by the UK Renal Registry

Routine clinical data collected by units for the Renal Registry returns will be transferred to the CTU for incorporation into the trial database annually (2017, 2018 and final download at study end) in the form of an electronic download (following appropriate testing procedures to ensure data integrity). This includes data collected for individual dialysis sessions (e.g. pre- and post-dialysis weights and blood pressures, dialysis prescription), haematology and biochemistry results, and treatment modality timelines, using the Renal Registry Dataset V4.2 (www.renalreg.org). If sites are not providing this information as part of their routine submission to the UKRR then they will be required to send a separate file with the fields by an appropriate secure mechanism to the UKRR at least once at the end of the trial. Admission and discharge dates, diagnostic and procedural codes will be obtained from Hospital Episodes and Statistics (or its equivalent body) by data linkage. If subjects choose to drop out of the study they will asked if they are still willing for their routine data be used (this is consented for independently).

### Statistical methods

Analysis will be in accordance with a predetermined statistical analysis plan and will be conducted blind to treatment allocation. Statistical significance will be set as *p* ≤ .05 (two-tailed).

#### Primary outcome

Time to anuria will be analysed on an intention-to-treat basis (as the primary analysis) and on an as-treated basis (as the secondary analysis) using competing risks survival analysis [32], to estimate the relative risk (as expressed by the sub-hazard ratio) of the outcome (anuria) in patients where BI is used compared to control patients, accounting for the competing risks (death, transplantation). Patients undergoing modality change or recovery will be censored at the point of treatment switch. The analysis will control for known baseline covariates affecting residual function [26, 29], i.e. age, race, sex, comorbidities (separately or using a validated scoring system), antihypertensive drug use (ACE inhibitors/ARBs, calcium antagonists) and diuretic use.

#### Secondary outcomes

Difference in rate of decline in renal clearance will be analysed using a random slopes linear mixed model, with adjustment for baseline characteristics, as for the primary outcome. We will analyse the effect of randomization on fluid status and body composition as determined by BI (to ascertain the effect of the intervention on the fluid assessment decision) and undertake corresponding appropriate analyses of the other secondary outcomes such as (i) *dialysis-related symptoms and treatment efficacy* (e.g. inter-dialytic fluid gain, falls, post-dialysis recovery time), (ii) *critical events* such as cardiovascular events and interventions, access-related interventions/failures and death, and (iii) *patient-reported measures* (e.g. EQ-5D-5 L, SF-12, PAM, POS-S renal, CSRI CKD). In analysing the effect of the intervention on patient activation measures we will look to see if this is associated with objective measures of fluid management, e.g. inter-dialytic fluid gain which, following adjustment for comorbidity, is a surrogate measure of patient survival.

#### Subgroup analyses

Pre-specified subgroup analyses will be limited to comorbid conditions that affect management of fluid status – specifically heart failure and diabetic status – and will be assessed through an interaction term in the model [33]. They will also explore, in a separate analysis, the effects of unit-level practice patterns (Table 2) as defined by our pre-study survey of 66 dialysis units, e.g. routine use of blood volume monitors, BI, dialysate sodium concentration, including stated approaches to fluid management (e.g. intention to reduce weight to avoid the use of antihypertensive drugs).

#### Economic evaluation

Economic analysis will be carried out to explore the relative cost-effectiveness of the intervention compared to standard management. The base-case analysis will adopt an NHS and personal social services perspective [34]. Additional analyses will be undertaken from a wider societal perspective, by considering private (patient-incurred) costs and productivity loss, using added questions from a modified Client Services Receipt Inventory developed for patients with chronic kidney disease. Costs and benefits accruing in the future will be discounted to reflect positive time preference.

A trial-based analysis will be carried out alongside the BISTRO study to determining the cost-effectiveness of the compared strategies on the basis of patient-level data obtained within the study period. Results will be presented in the form of incremental cost-effectiveness ratios, reflecting the extra cost for an additional unit of outcome. The main outcome will be the QALY, determined from participants’ responses to the EuroQol EQ-5D-5 L [21] and SF-12 [35] instruments at baseline and 3-monthly thereafter. To account for uncertainty, the inherent uncertainty due to sampling variation, the joint distribution of differences in cost and outcomes (QALYs) will be derived by carrying out a large number of non-parametric bootstrap simulations [36]. Results will be depicted on a cost-effectiveness plane and will be plotted as cost-effectiveness acceptability curves (CEACs) [37]. CEACs will show the probability of the BI-guided and standard fluid management options being cost-effective across a range of possible values of willingness to pay for an additional QALY. Additional work will involve building a decision analytic model to assess costs and effects likely to accrue beyond the study follow-up period**.**

### Monitoring

The Keele CTU data management team will perform data quality checks of CRF data. Data queries will be entered to a log that will be sent to the trial manager, who will work with each site to resolve data queries in a timely fashion and provide further training as required. This, along with safety reports, will inform a risk-based approach towards assessing a need for any onsite monitoring visits. Trial monitoring reports will be reviewed by the Trial Management Group, Data Management Committee (DMC) and Trial Steering Committee (TSC). These committees are constituted according to NIHR Health Technology and Assessment Board guidelines and are fully (DMC) or >75% independent (TMC). The DMC will receive un-blinded safety data at the group level and the TMC will receive reports from the DMC.

## Discussion

Adoption of new technologies to support clinical management requires scrutiny no less than the introduction of new pharmacological interventions. Bioimpedance is not new, although the technology has become more sophisticated, and the actual process of taking measurements is both safe and reproducible in trained hands [12]. Introduction of this technology to the clinical setting has been found to be acceptable to patients and many dialysis units around the world are now undertaking regular measurements as part of clinical practice. The evidence that BI gives clinicians new information about their patients’ body composition is strong and it is increasingly clear that the combination of reduced lean tissue mass and excess extracellular water (ECW) in proportion to intracellular water (ICW) – however this is expressed (phase angle, ECW/ICW, overhydration index) –, is associated with worse clinical outcomes.

The question remains how to use this additional information in day-to-day clinical management. Should clinicians attempt to normalize measured ECW to the patient’s normally hydrated weight or should they deliberately volume-deplete patients by the end of their dialysis treatment in an attempt to control blood pressure and undo the damage associated with long-standing hypertension? Without an independent measure of body composition, how do clinicians know which of these strategies they are employing? Given the potential to cause harm by excessively and serially volume-depleting patients, it would seem crucial that this practice is properly evaluated in a clinical trial. BISTRO is designed to fill this knowledge gap, focussing on the simplest of potential casualties of volume depletion, residual kidney function, a commodity that has been shown to be of consistent benefit to dialysis patients.

Trials assessing fluid management are challenging to undertake. One of the reasons for this is that assessing fluid status and implementing change are an example of complex decision making. The clinician, working with the patient, needs to take several things into account (e.g. symptoms, dietary intake, multiple comorbidities, dialysis tolerance, conflicting diagnostic information) and choose from several interventions (e.g. salt restriction, increased or reduced fluid removal, pharmacological interventions, and often more than one). One of the lessons learned from the UK-Shanghai BI trial in peritoneal dialysis patients [17], which attempted to collect decisions made at the time of fluid status assessment, was just this –these decisions are complex, multiple and bi-directional over time. To try and understand this better for BISTRO we have developed a template that will both help clinicians order their approach to fluid assessment and clarify why they chose to over-ride the BI-derived normally hydrated weight. The disadvantage of this approach may be that fluid management is ‘improved’ in the control group, so putting the intervention under greater pressure to demonstrate a benefit; the advantage is a better understanding of the intervention and even if the study is negative we will have, for the first time, a validated template to support fluid management.

Performance bias is also a risk in this type of study, which has inevitably to be an open-label design. We have endeavoured to minimize this risk by separating the BI measurements from the clinical assessments and blinding both clinicians and participants to the BI measures in the control group. This will be a major focus of the training sessions that will be undertaken at all the sites, which will also ensure consistency in how the intervention is implemented.

Finally, in designing the trial we carefully considered the pros and cons of a cluster (dialysis unit) versus a patient-level randomisation. Apart from practical reasons (for example patients often transfer from the main dialysis unit to a satellite centre after initiation of treatment, potentially forcing either drop in or out of the intervention) we became aware of significant variation between sites in their approach to fluid management. We undertook a survey of UK dialysis units to establish this, confirming that the number of sites required to get a balanced cluster randomization would have been impractical [11]. We have, however, used the information from this survey to undertake an annual survey of practice patterns during the conduct of the trial to allow us to correct for these in the primary and secondary analyses. Understanding the interactions between these practice patterns and clinical outcomes are an additional, potentially useful benefit of the study.

## Abbreviations

BCM: Body composition monitor
BI: Bioimpedance
BISTRO: Bioimpedance spectroscopy to maintain renal output (Trial acronym)
CANUSA: Canada USA study
CEAC: Cost-effectiveness acceptability curves
CRF: Clinical report form
CSRI: Client service receipt inventory
CTU: Clinical trials unit
DEXA: Dual energy X-ray absorptiometry
DMC: Data monitoring committee
ECF: Extra-cellular water
EQ-5D-5 L: European quality of life (Qol) 5 dimension 5 level score
GFR: Glomerular filtration rate
HD: Haemodialysis
ICW: Intracellular water
IPOS: Integrated palliative care outcome scale
MoCA: Montreal cognative assessment tool
NECOSAD: Netherlands cooperative study of adequacy in dialysis
PAM: Patient activation measure
QALY: Quality-adjusted life years
RKF: Residual kidney function
SF12: Short form 12 (Health Survey)
TSC: Trial steering group
